# Exploring the usefulness of medical clowns in elevating satisfaction and reducing aggressive tendencies in pediatric and adult hospital wards

**DOI:** 10.1186/s12913-020-05987-9

**Published:** 2021-01-06

**Authors:** Dorit Efrat-Triester, Daniel Altman, Enav Friedmann, Dalit Lev-Arai Margalit, Kinneret Teodorescu

**Affiliations:** 1 Ben-Gurion University of the Negev, 8499000 Beer-Sheva, Israel; 2 Technion – Israel Institute of Technology, Haifa, Israel; 3 The Academic College of Tel Aviv-Yaffo, Tel Aviv-Yaffo, Israel

**Keywords:** Medical clowns, Health care management, Health consumer satisfaction, Aggression, Age

## Abstract

**Background:**

Most existing research on medical clowns in health care services has investigated their usefulness mainly among child health consumers. In this research we examined multiple viewpoints of medical staff, clowns, and health consumers aiming to identify the optimal audience (adult or child health consumers) for which medical clowns are most useful. We focused on exploring their usefulness in enhancing health consumers’ satisfaction and, in turn, reducing their aggressive tendencies.

**Methods:**

We conducted three studies that examined the placement fit of medical clowns from different points of view: medical staff (Study 1, *n* = 88), medical clowns (Study 2, *n* = 20), and health consumers (Study 3, *n* = 397). The main analyses in Studies 1 and 2 included frequencies and t-tests comparing perceived adult and child satisfaction with clowns’ performance. Study 3 used moderated-mediation PROCESS bootstrapping regression analysis to test the indirect effect of negative affectivity on aggressive tendencies via satisfaction. Exposure to the medical clown moderated this relationship differently for different ages.

**Results:**

Studies 1 and 2 show that the majority of medical clowns and medical staff report that the current placement of the medical clowns is in pediatric wards; about half (44% of medical staff, 54% of medical clowns) thought that this placement policy should change.

In Study 3, data from health consumers in seven different hospital wards showed that clowns are useful in mitigating the effect of negative affectivity on satisfaction, thereby reducing aggressive tendencies among health consumers under the age of 21.6 years.

Surprisingly, medical clowns had the opposite effect on most adults: for health consumers who were exposed to the medical clown and were above the age of 21.6 negative affectivity was related to decreased satisfaction, and an increase in aggressive tendencies was observed.

**Discussion:**

Medical clowns are most useful in elevating satisfaction and reducing aggressive tendencies of children. Older adults, on the other hand, exhibit lower satisfaction and higher aggressive tendencies following exposure to the performance of medical clowns.

**Conclusion:**

Medical clowns should be placed primarily in children’s wards.

**Supplementary Information:**

The online version contains supplementary material available at 10.1186/s12913-020-05987-9.

*“The role of a clown and a physician are the same - it's to elevate the possible and to relieve suffering.”*

*Patch Adams*

## Background

The use of clowns as a service in hospitals is taken from the circus world and is applied to contexts of illness, to improve people’s mood and state of mind, as well as to promote health consumer satisfaction and compliance [1]. Evidence for the existence of clowns in hospitals goes back to Hippocrates [2], or even prehistoric times [3, 4]. Yet, it seems that only after the premiere of the movie Patch Adams in 1998, did people become more aware of the potential benefits of medical clowns (e.g., humor and laughter create an atmosphere of trust and love between staff and health consumers) [4, 5]. While Patch Adams referred to clowning for a variety of audiences, most medical clowns operate in pediatric wards, and accordingly, most studies focus on child health consumers [6]. It has recently been suggested that medical clowns can also benefit a variety of health consumers with different health problems, including adults, in line with Adams’ approach [7–9].

A key precursor of purchase intentions for health services is consumer satisfaction [10], and so, just like other service organizations, health services must seek the satisfaction of their health consumers [11]. Consumer satisfaction with the service a hospital provides is the sense a customer has that the hospital fulfills some need, desire, goal or so forth and that this fulfillment is pleasurable, or in other words, “satisfaction is the health consumer’s sense that the hospital provides outcomes against a standard of pleasure versus displeasure” ( [12] , p.80). Service satisfaction indicates the extent to which the overall service that health consumers receive is congruent with their expectations, and so it is a subjective measure [13]. As such, satisfaction contains the person’s appraisal, which includes both affective (or emotional) and cognitive (evaluative) components [14, 15]. Health consumer satisfaction is a unique structure, distinct from regular customer satisfaction since healthcare services have a major impact on physical wellbeing [16, 17]. Hence, a bad service experience in the healthcare context is more threatening than a bad service experience in general, as it might constitute a life threat [18]. The experience of health consumers creates satisfaction and usage of the service [19], and a clown can influence that experience [20, 21]. Specifically, the medical clown is an added service and part of the medical team that provides various “service” treatments for health consumers in specific wards [22, 23]. Health consumers’ satisfaction with each member of the medical team, which consists of several specialists, including their satisfaction with the medical clown, can influence their overall satisfaction with the hospital [24, 25]. Medical clown services are specifically known to have an impact on the satisfaction of health consumers with the hospital in general [26]. The current study draws from literature in the fields of service management and organizational behavior to better understand the influence of medical clowns on the perceived satisfaction of health consumers in different hospital wards. This exploration is important as health consumers’ satisfaction measures can help identify the optimal health care audience (adults or children) that will most benefit from medical clowns.

The use of medical clowns in hospitals is a growing phenomenon, used as a therapeutic method in addition to traditional medical practices and as a negative affectivity buffer [27]. Previous research shows that medical clowns have a positive impact on the physical and psychological well-being of health consumers [25, 28, 29], reducing the need for pain medications [30], lowering negative affectivity levels [31, 32], increasing positive feelings [20, 33, 34], and enhancing health consumer satisfaction with the hospital service [1]. The positive effect of medical clowns on both health consumers and medical staff can improve the outcomes of medical interventions [16, 20].

Clown usefulness can be measured by various measurements including physical well-being, psychological well-being, negative affectivity, levels of positive feelings, and overall satisfaction [18–26]. As satisfaction plays an important role in physical and psychological well-being [35, 36], it will be the focus of this paper in which we will explore medical clown usefulness as an indication of the optimal placement fit.

Most findings on medical clowns are based on separate children or adult samples, rather than on integrated samples, and the placement fit of the medical clown versus audience age has not been addressed (e.g. only adult wards: [8, 33, 34]; only pediatric wards: [25, 28, 32, 37]. Medical clowns are usually placed in pediatric wards [38], and so the majority of research on medical clowns is based on children samples. Since the presence of medical clowns in adult wards has increased substantially in the past decade, especially with older [27], and chronic health consumers [24], more research examining the unique effect of medical clowns on adults vs. children is necessary.

The literature on the effect of medical clowns on adults is still in its infancy. The adult audience is insinuated as being less suitable for the services of medical clowns, as clowns reported both a need for greater creativity when working with adults compared with children, and more strain and feelings of frustration due to the effort required to continually make older people laugh [24]. Others, however, have suggested that medical clowns may benefit adults as well [8], especially when considering specific individual differences in the receptiveness to humor [39]. The literature on the effect of medical clowns on adults needs further investigation [26].

In the current paper, we aim to fill this gap by comparing the usefulness of medical clowns among adult and child audiences and identifying the optimal placement of medical clowns.

In order to identify the optimal placement of medical clowns, we first identify the current placement of clowns in hospital wards, examine the medical staff’s perceptions regarding the usefulness of clowns among current audiences, and document the clowns’ experience of performing to both types of audiences. As managers in healthcare prioritize implementation processes [40], we aim to provide policy recommendations regarding the best placement fit of the medical clown. Building on previous literature according to which most clowns are placed in pediatric wards [27, 41] and most pediatric health consumers are satisfied with the clowns’ performance [1], we expect that medical staff and clowns will report that pediatric child health consumers are more satisfied with clowns than are adult health consumers. Hence, we predict:

- *H1a.* Medical staff will report that medical clowns will increase the satisfaction of children more than that of adults.
- *H1b.* Medical clowns will report that medical clowns will increase the satisfaction of children more than that of adults.

### Health consumer satisfaction and aggressive tendencies

Hospitals are a stressful environment for health consumers [42], leading them to a state of emotional negativity and dissatisfaction [43, 44]. In the hospital context, dissatisfaction is considered a central cause of health consumer aggression against medical staff [45]. We define aggression, as per Rippon [46], as “a behavior with intent that is directed at doing harm to a living being whether harm results or not … aggression can be physical or verbal, active or passive, and can be focused on the victim(s) directly or indirectly” (p. 456). Aggressive behavior of health consumers towards medical staff in hospitals is a dangerous global problem that has potentially detrimental outcomes for staff [47–49], as well as high financial costs for the hospitals [50]. This phenomenon is therefore labeled as an “epidemic” [51], which constitutes an occupational hazard [52]. Health consumer aggression against medical staff affects all parties involved [53], ranging from the targeted staff who suffer verbal and physical abuse, to other health consumers who receive medical care from burnt-out staff with depleted cognitive resources [54], and the hospital that suffers from high staff turnover [47].

Health problems are considered among the top ten most stressful life events [55, 56]. When an illness is serious enough to warrant an individual’s confinement to a hospital, the mere process of hospitalization may produce even greater negative affectivity [57]. Individuals who exhibit high negative affectivity tend to show distress, sensitivity to negative events, and a pessimistic view of events and their surroundings [58, 59]. When negative affectivity increases, individuals report lower satisfaction (for a meta-analysis, see [60]). In a meta-analysis examining the relationship between negative affectivity and satisfaction, in a different context, negative affectivity was consistently related to lower satisfaction [61]. Specifically, this result was found when examining job satisfaction and not consumer satisfaction with a service, even though “satisfaction” shares the same definition of having an emotional and cognitive evaluation of an object (whether it is a job, a service, or even life as a whole) [14, 15].

Indeed, negative affectivity was found to be correlated with service satisfaction specifically [62]. Other scholars suggested that the negative affectivity of health consumers or their families (caused by a variety of reasons) is directly related to aggressive tendencies in the hospital setting [63, 64]. We define aggressive tendencies following [65] as the self-reported desire to act with aggression. We chose this focus since the desire to act with aggression is an important predictor of actual aggressive behavior [65], and as such, it is important to curtail this desire before it escalates into actual aggression. Aggressive tendencies can be expressed in a variety of forms, from interpersonal conflict to bullying or even physical assault [66]. In hospitals, this aggression is frequently targeted towards those trying to help others, i.e. the medical staff. Research aimed at understanding aggression as a consequence of satisfaction, in service organizations in general, and in hospitals in particular, is scarce [51], even though an understanding of this issue would help in developing active attempts to curtail aggression.

Health consumer satisfaction is a product of the interaction between health consumers and medical staff [67]. Although health consumers arrive at the hospital with expectations of relieving their pain and illness and improving their overall wellbeing [68], hospitalization raises negative affectivity in general, and anxiety in particular [69]. Negative affectivity includes a spectrum of negative emotions, ranging from anger to fear, to annoyance and anxiety [59]. These negative emotions paint the health consumers’ experience [19, 70] and lower their satisfaction with the service organization [71], which if low, can even elicit their tendency to behave aggressively. As the overall goal of the study was to identify the optimal health care audience (adults or children) that will benefit most from medical clowns, we examined specifically whether medical clowns can buffer the harmful effect of negative affectivity on health consumer satisfaction, and whether this buffering effect depends on the age of the health consumer audience. We propose satisfaction as the underlying mechanism that explains why negative affectivity influences aggression:

- *H2.* Health consumer satisfaction mediates the indirect relationship between health consumers’ negative affectivity and their aggressive tendencies towards medical staff.

### Medical clowns as moderators of the relationship between negative affectivity and satisfaction

A practice commonly used to enhance health consumer satisfaction is the use of medical clowns in hospital wards. As noted, hospital wards are characterized by high stress, negative affectivity, and aggression [42, 57], yet very little research has been done to reduce such aggression. The few attempts that have been made, such as [72], focused on providing health consumers with information to enhance perceived justice, and did not focus on elevating their satisfaction. As a result, the emotional state of the health consumers was never fully examined in this context. In the current study, we aim to fill this gap by empirically testing the medical clowns’ ability to buffer the harmful effect of negative affectivity on satisfaction. We predict that medical clowns have this ability since they are known to create a positive mood and change people’s negative state of mind to more humorous, thus promoting health consumer satisfaction [1, 19, 20].

Most research on medical clowns focuses on children, showing the positive effect of medical clowns on young health consumers [21, 27]. For example, medical clowns reduced stress among 6–7 years old, as was evident in a study that measured children’s cortisol levels following interaction with a medical clown [31]. Moreover, medical clowns reduced pain, crying, and anxiety in children aged 2–10 years old undergoing venous blood drawing [37]. A systematic review of medical clowns in pediatric wards shows their positive influence in reducing anxiety and pain [73].

Far less research examined the influence of medical clowns on adult populations, and the few studies that did, found positive effects [8, 24, 33, 34]. Strikingly, these studies examined either children or adult samples, separately, without comparing the two age groups or examining the placement fit of the medical clown to audience age. Indeed, humor and laughter are known to have a positive effect on both populations [74, 75], and research that empirically tests the unique effect of medical clowns on adults versus children is necessary. In the current paper, we aim to fill this gap by comparing the usefulness of medical clowns among these audiences and identifying the optimal placement of medical clowns.

We predict that medical clowns affect both populations positively, with a stronger positive influence on children, due to the common placement of clowns in pediatric wards, the large body of research examining pediatric health care consumers, and the tendency of children to seek play and humor [76]. We therefore predict:

- *H3.* The usefulness of medical clowns in enhancing health consumer satisfaction and, in turn, in reducing aggression, depends on the health consumers’ age, such that the positive effect of clowns on the relationship between negative affectivity and satisfaction will be stronger among children compared with adult health care consumers.

See Fig. 1.

**Fig. 1:**
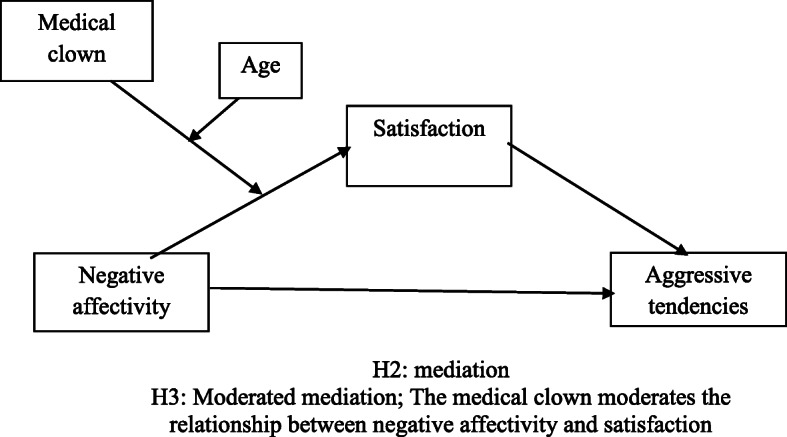
Theoretical model of Study 3: The relationship between negative affectivity and aggressive tendencies via satisfaction is moderated by the medical clown and patient age

### Overview of studies

Using a multiple views research approach, we examined the optimal health consumer audience for medical clowns. Study 1 examined the perceptions of medical staff regarding the audience that would be most satisfied by the clowns. Study 2 examined the perception of the medical clowns regarding their optimal placement.

Study 3 examined the optimal placement of the medical clown from the health consumers’ point of view, with both child and adult health consumer participants. In this last study, we examined the medical clown’s usefulness in enhancing health consumers’ satisfaction and, in turn, reducing their aggressive tendencies.

### Availability of data and materials

Data can be found at https://osf.io/8j7ar/?view_only=c0cd6308be634b948235132df01612bd

## Study 1: medical staff

### Method

#### Participants and procedure

Data were collected from 88 medical staff members while attending a health care management course as part of a health care science MBA degree at a university in southern Israel. The subjects completed a short (5 min) paper-and-pencil survey, during their class participation. Participation was voluntary. Data were kept anonymous, and answers to the survey did not reveal the participants’ identity. The class grade was not affected in any way by completing/not completing the survey. The survey and method of data collection were approved by the university’s ethics committee. Data collection took 1 day. (Mage = 37.35, sd = 7.08; 75% women; Mtenure = 11.38 years, sd = 7.59).

Of the medical staff, 25% were health care managers (in various professions: doctors, nurses, etc.). The medical staff’s professions were as follows: 12% were doctors, 61% were nurses, 7% were X-ray technicians, 10% were paramedical professionals (physical therapists, dieticians, speech therapists), and the remaining 10% preferred not to report their profession.

#### Measures

*Current placement policy* was measured by asking the medical staff where medical clowns are currently placed.

*Perceived optimal placement policy* was measured by asking the medical staff whether the current policy regarding the medical clowns’ placement should be changed, using the open-ended question “What is your opinion about the current ward where the medical clowns are placed in your hospital?” Answers were coded by two judges, as follows: 1 - no change to placement policy needed; 2 - change the placement policy so that they are placed more frequently in adult wards; 3 - change the placement policy so that they are placed more frequently in pediatric wards; 4 - change the placement policy so that clowns are generally placed more frequently in all wards; and 5 - change the placement policy so that clowns are placed less frequently in general.

*Perceived health consumer satisfaction with the clowns* was measured based on Wong’s [15] definition of satisfaction, which includes both affective (or emotional) and cognitive (evaluative) components. Specifically, we based the measurement on happiness and satisfaction items adapted from [77], adjusting these items to the specific question of satisfaction with the medical clown, as follows: “To what extent are most adults satisfied/happy with the clown’s performance; To what extent are most children satisfied/happy with the clown’s performance” (Cronbach’s alpha = .83).

#### Demographics

We asked participants their age, gender, job title, and tenure in their current job.

The survey developed for this study is provided as Additional File 1.

#### Statistical analysis

We examined first descriptive statistics regarding current placement policy and perceived placement policy. To test H1a, which predicted that medical staff will report that medical clowns will increase the satisfaction of children more than that of adults,

we performed a paired sample t-test to compare how medical staff perceived adult and child satisfaction with medical clowns.

### Results

#### Current placement policy

Most medical staff (63%) reported that medical clowns are placed only in pediatric wards, 24% reported they are placed in both pediatric and adult wards, 8% of medical staff reported that their organization currently does not employ any medical clowns.

#### Perceived placement policy

Most medical staff (64%) perceived the current policy placement of medical clowns as adequate. The remaining 36% thought the current policy should be changed. The recommended policy change was distributed as follows: 44% recommend placing more clowns in pediatric wards, 54% recommended placing more clowns in adult wards or in all wards (16% in adult wards; 38% in all wards). Only 2% reported that they would decrease the number of clowns in hospitals, in general.

#### Perceived health consumer satisfaction

T-test results indicate that the medical staff perceived children’s satisfaction with the clowns as significantly higher (M = 6.13, sd = 1.19) than that of adults (M = 4.19, sd = 1.62; t [78] = 10.91, *p < 0.001*), confirming H1a.

After examining the medical staffs’ reports on clown placement and perceived health consumer satisfaction following clown performance, we turned to examine the medical clowns themselves. Specifically, we were interested in asking the clowns whether they adapt their performance to different health consumer audiences, what criteria they use to adapt their performance, and how they perceive consumer satisfaction following their performance.

## Study 2: medical clowns

### Method

#### Participants and procedure

Data were collected from 20 medical clowns who participated in an online survey. In order to recruit the medical clowns, we contacted the Israeli National Medical Clown Organization. We invited them via email to complete a short (10 min) online survey, in return for participation in a lottery for a breakfast voucher. The survey and data collection method were approved by the university’s ethics committee. Data were kept anonymous, and answers to the survey did not reveal the medical clowns’ identity in any way. Data collection took 1 week. (Mage = 40.68, sd = 6.10; 50% women; Mtenure = 8.84 years, sd = 4.93). All clowns worked in Israeli hospitals. Most clowns (65%) worked in a single hospital and 35% worked in two hospitals. Most of the clowns worked as clowns only part-time (85%), and 64% of the part-time clowns had a second job as theater actors.

### Measures

*Audience fit of clowns’ performance* was measured by asking the medical clowns whether they adapt their performance to the audience type, and if so, what criteria they use (age, ward type, medical condition, gender, language).

*Current placement policy* was measured by asking the medical clowns where they are currently placed.

*Perceived placement policy* was measured by asking the clowns about their perspective on the hospital policy regarding their placement in different wards, as described in Study 1.

*Perceived health consumer satisfaction with the clowns* was measured using the same measure as in Study 1, adapted to medical clowns. Cronbach’s alpha = .89.

#### Demographics

we asked participants their age, gender, job title, and tenure in their current job.

The survey developed for this study is provided as Additional File 2.

### Statistical analysis

We examined first descriptive statistics regarding audience fit of clowns’ performance and current and perceived placement policy. To test H1b, which predicted that medical clowns will report that they will increase the satisfaction of children more than that of adults, we performed a paired sample t-test to compare how medical clowns perceived adult vs. child satisfaction with medical clowns.

### Results

#### Audience fit of clowns’ performance

Most of the clowns (95%) reported that they adapt their show to the audience, according to the following criteria: age (85%), medical condition (80%), gender (55%), ward type (50%), and language (55%).

#### Current placement policy

Most clowns (60%) are placed only in pediatric wards, and 40% are placed in both pediatric and adult wards.

#### Perceived placement policy

About half (54%) answered that they would change the policy regarding their placement to be placed more frequently in adult wards. 15% would not change the placement policy, and 30% answered that they would place more clowns in hospitals, in general.

### Perceived health consumer satisfaction with the clowns

Paired sample t-test results indicate that the clowns perceived the children’s satisfaction (M = 6.63, sd = .48) as significantly higher than the adults’ satisfaction (M = 6.08, sd = .71; t [19] = − 5.09, *p < 0.001*), confirming H1b.

Studies 1 and 2 examined the medical staff’s and clowns’ perceptions regarding the ability of medical clowns to enhance health consumer satisfaction among different health consumer audiences. Both studies identified the clowns’ perceived optimal audience (i.e. children). Study 3 continued Studies 1 and 2 and used a hospital field study to explore the actual optimal placement of medical clown, comparing adult and child health consumer satisfaction following a visit of a medical clown. Furthermore, Study 3 broadens the scope of Studies 1 and 2 by revealing the antecedent, moderators, and consequence of health consumer satisfaction as a way to explore the medical clown’s usefulness.

## Study 3: health consumers

In order to explore the most useful fit for the medical clown placement, Study 3 aims to achieve three goals: (a) Determine the underlying mechanism that influences health consumers’ aggression towards medical staff, (b) investigate the usefulness of medical clowns as an intervention aimed at increasing satisfaction and reducing aggression, and (c) determine which target health consumer audience can benefit the most from this intervention.

### Method

#### Participants and procedure

The study took place in a large hospital located in northern Israel. The project was approved by the hospital’s board committee and monitored by the hospital’s research authority. Research assistants approached health consumers and asked them to participate in a voluntary and anonymous study about the service they received at the hospital. Participants gave oral consent and signed an informed consent form according to the hospital’s research authority regulations. The inclusion criteria were as follows: children - aged 5–18, being accompanied by a family member, and having parental or legal guardian’s consent to participate; adults – being mentally healthy, and understanding the survey. Children under the age of 5 were not included since it was thought that they may lack the skills necessary to report their own thoughts and emotions and may misunderstand the response scale options used (following [79]). Children who needed assistance reading and answering the survey received parental help.

Data were collected from a convenience sample of 387 health consumers, in a between-subject field study. Participants were sampled at random hours and days of the week and in seven hospital wards (Children wards: Pediatrics A and B, Surgery, and Oncology; Adult wards: Dialysis, Hematology, and Oncology) to simulate a random sample as much as possible, in the field.

Thirty-three percent of participants were treated ambulatorically, 59.8% were women/girls, and 72.8% were adults. The mean age of children was 11.83 (sd = 3.82) and the mean age of adults was 40.26 (sd = 13.88). The mean years of education for adults were 13 (sd = 2.46). The native tongue for 42.8% of the sample was Hebrew, for 48% it was Arabic, and 4.8% reported that Russian was their native tongue.

One hundred and thirty-eight participants (36.3%) observed a medical clown performance, when he/she came to visit them in their ward, the same day or the day before completing the survey, and constituted the treatment group, while the rest of the health consumers served as a control group.

The time frame for answering the questionnaire was based on the finding that the effect of the clown on health consumer’s negative affectivity is detected, in most studies, up to 1 day later [20].

Random assignment to conditions is assumed since (a) medical clowns arrived at the hospital on random days and hours, (c) clowns’ route within the hospital was random as well, and (c) our research assistants arrived at the hospital on random days and hours. Health consumers were invited to voluntarily participate in a study about the quality of service in their department by responding to a survey (available in the three prevalent languages in Israel: Hebrew, Arabic, and Russian). After responding to our survey, participants were thanked and given a small bar of chocolate as a token of appreciation for their participation.

The survey was composed of items that were all previously published elsewhere, as described below. Items were translated and back-translated from English into Hebrew, Russian, and Arabic.

#### Measures

*Negative affectivity* was measured using nine items from the PANAS, developed by Watson, Clark, and Tellegen [80]. Participants were asked to rank, using a 7-point Likert-type scale, the degree to which they feel certain emotions in the current moment. Sample items included: “annoyed”, “stressed”, “angry”. Answers ranged from 1- not at all to 7- very much. Cronbach’s α = 0.89.

*Aggressive tendencies* were defined as the self-reported desire to act with aggression [65]. The measure of aggressive tendencies was based on [72], focusing on four aggressive tendencies that fit the context of the large variety of wards in which the medical clowns performed. Specifically, we asked participants to evaluate the likelihood that in the near future someone in their ward will curse the medical staff’s family, vandalize hospital property, bang on a table, or complain about medical staff. Answers ranged from 1- very low to 7- very high. Cronbach’s α = 0.69.

*Satisfaction* with the medical team and hospital was measured based on Wong’s definition and Allik’s happiness and satisfaction items after being adapted for the team and the hospital as a whole [15, 77]. However, unlike Study 1 and 2, satisfaction was measured with respect to the medical team and the hospital in general and not only with respect to the clown’s performance. Participants were asked to evaluate, using a 7-point Likert-type scale, the extent to which they are satisfied with the treatment provided by the medical team and the extent to which they are satisfied with the hospital. Answers ranged from 1- very dissatisfied to 7- very satisfied. Cronbach’s α =0.67.

#### Medical clown

The operational definition of a medical clown is an intervention strategy for therapeutic treatment of health consumers in hospitals [81]. They are trained professional performers who use techniques such as magic, music, storytelling and other clowning skills to entertain health consumers [5]. Medical clowns are high on personal qualities, such as empathy, emotional intelligence, and intuition [8] and come from a variety of backgrounds—clowning, acting, physical theatre, mime, music, close-up magic. Hospital clowning is not sustainable on a full-time basis and is usually combined with their original type of performing. Medical clowns are an integral part of the medical staff, with clowns making rounds, like doctors, in the ward in which they are positioned per hospital policy [82].

Participants reported whether or not they encountered a medical clown, and if so, when. Answers were such that if they met a clown on the day they answered the survey or on the day before, the answer was coded “yes”, and if not, it was coded “no” (dichotomous measure). This question was asked in order to determine whether or not the medical clown had an impact on the participants and is in line with recommendations that cognitive and affective processes last 2 days [78].

#### Demographics

Participants were asked to indicate their age, gender, years of education, religion, mother tongue, and social-economic status (calculated by dividing the number of people in the home by the number of rooms in the home).

#### Statistical analysis

We first performed an independent sample t-test to ensure no evidence of differences in demographic variables across conditions of encountering/not encountering a clown in the last 2 days. Then, exploratory factor analysis with a varimax rotation was used to test the divergent validity of negative affectivity, aggressive tendencies, and satisfaction.

Descriptive statistics of the main variables of this Study 3 were analyzed. Regression analyses were conducted to explore the relationship between negative affectivity and health consumers’ aggressive tendencies; negative affectivity and satisfaction with their treatment; and satisfaction and tendency to engage in aggressive behavior. We further tested the mediation model (Hayes [83], Model 4), and the role of satisfaction in mediating between negative affectivity and aggression tendencies to test H2. To test H3, which assumed that the usefulness of medical clowns in enhancing health consumer satisfaction and, in turn, in reducing aggression, depends on the health consumers’ age, we used Hayes’ moderated mediation model (Model 11). In this analysis, we expect that the positive effect of clowns on the relationship between negative affectivity and satisfaction will be stronger among children compared with adult health care consumers.

### Results

To assure sample compatibility (randomness of respondents under the different study conditions of encountering/not encountering a clown in the last 2 days), we compared the demographic variables of respondents (age, years of education, religion, mother tongue, and social-economic status) across conditions. No evidence of differences in demographic variables across conditions was found, indicating that the samples are indeed comparable (T*Age* (367) = − 1.8, *n.s*; T*Eduacation* (359) = 0.004, *n.s*; T*Religion* (372) = 0.93, *n.s*; Chi square [*Mother Tongue*] = 6.38, *n.*s;TMother *Tongue* [370] = − 1.23, *n.s;* T*Social-Economical status* [365] = − 1.52, *n.s*).

Exploratory factor analysis confirmed three factors, with separate factors corresponding to the following variables: negative affectivity, aggressive tendencies, and satisfaction. The Kaiser-Meyer-Olkin measure of sampling adequacy was satisfactory and equaled 0.86. Bartlett’s test of sphericity was significant (Chi-square = 1723.69; *p* < .001). Table 1 presents means, standard deviations, and intercorrelations of Study 3 variables.

**Table 1:** Means, standard deviations, and intercorrelations of Study 3 variables

	***M***	***SD***	1	2	3	4
1. Negative affectivity	2.35	1.26	–			
2. Satisfaction	4.08	0.89	−0.25**	–		
3. Aggressive tendencies	1.81	1.10	.281^**^	−0.33**	–	
4. Age	32.45	17.45	−0.01	0.02	−0.03	–
5. Medical clown	0.36	0.48	−0.03	−0.04	− 0.04	−0.09

** *p* < 0.01

Regression analyses examining the influence of negative affectivity verified that negative affectivity increases health consumers’ aggressive tendencies (*β* = 0.25; *p* < 0.001), and that negative affectivity reduces health consumers’ satisfaction with their treatment (*β* = − 0.17; *p* < 0.001). Regression analysis also confirmed that the higher their satisfaction, the lower their tendency to engage in aggressive behavior (*β* = − 0.41; *p* < 0.001).

**Fig. 2:**
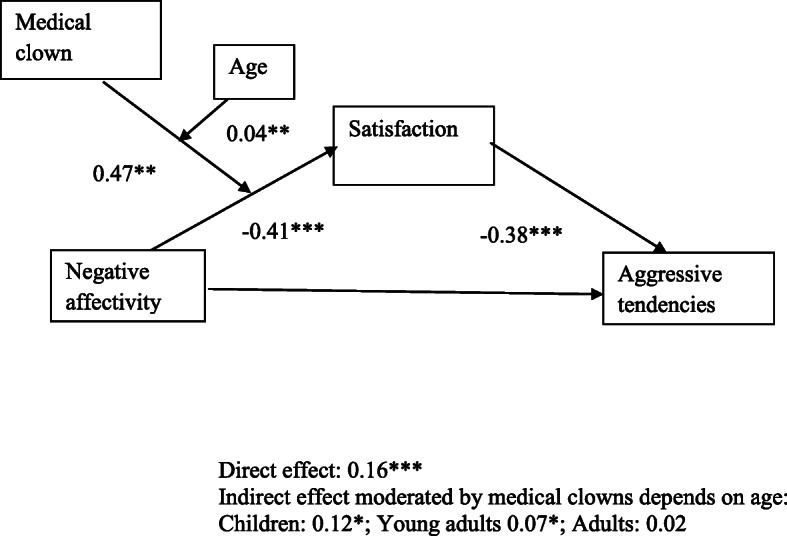
Results of Study 3: The relationship between negative affectivity and aggressive tendencies via satisfaction is moderated by the medical clown and patient age (Moderated-mediation, Process model 11)

H2 was tested using a classic mediation model developed by Hayes ( [83], Model 4) and the relationship between negative affectivity and aggression was mediated by satisfaction. As Fig. 2 illustrates, the standardized regression coefficient between negative affectivity and satisfaction was significant, as was the standardized regression coefficient between satisfaction and aggression. The standardized indirect effect was Indirect = 0.06, CI, 95%, [− 0.17, − 0.34]. We tested the significance of this indirect effect using bootstrapping procedures. Unstandardized indirect effects were computed for each of 5000 bootstrapped samples (CI, 95%, [0.03, 0.1]). In other words, the indirect effect was statistically significant (R^2^ = 0.06, *p* < 0.001). (See Fig. 2).

**Fig. 3:**
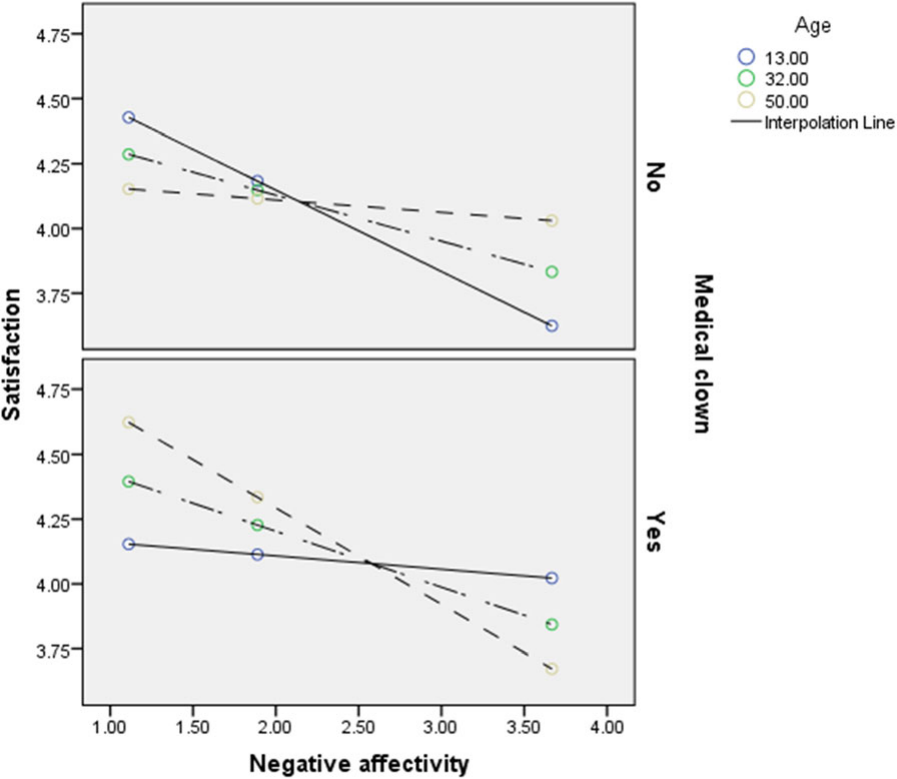
Graphical results of Study 3: The relationship between negative affectivity and satisfaction is moderated by medical clown and patient age

H3 was tested using Hayes' moderated mediation model (Model 11) and was fully supported. The indirect influence of negative affectivity on aggression (R^2 =^ 0.1, *p* < 0.001) was mediated by satisfaction. The moderating effect of medical clowns on the relationship between negative affectivity and satisfaction was dependent on age. A Johnson-Neyman significance region analysis [78] showed that for health consumers who *did not encounter* a medical clown, negative affectivity decreased satisfaction significantly for health consumers under 43 years old, and had no significant effect above that age. However, for health consumers *who encountered a clown*, the effect of negative affectivity on satisfaction was not significant under the age of 21.6, and significant above that age. This means that medical clowns successfully mitigated the indirect effect of negative affectivity on aggression for children and young adults under the age of 21.6. Results support our prediction that medical clowns buffer the harmful indirect effect of negative affectivity on aggression via satisfaction among children. Surprisingly, the medical clowns had a harmful effect on most adults. See Table 2. Fig. 3.

**Table 2:** Regression analysis testing predicted effects of negative affectivity, age and medical clown appearance, on health consumers’ satisfaction and aggressive tendencies in Study 3 (Moderated-mediation, Process model 11)

	*Dependent variable*			
	*Satisfaction*		*Aggressive tendencies*	
Constant	4.98***	(0.26)	2.99***	(0.30)
Negative affectivity	−0.41***	(0.10)	0.16***	(0.04)
Age	−0.02*	(0.01)		
Medical clown (0 = no, 1 = yes)	−1.06**	(0.38)		
Satisfaction			−0.38***	(0.06)
Negative affectivity x Age	0.01*	(0.00)		
Negative affectivity x Medical clown	0.47**	(0.15)		
Age x Medical clown	0.04**	(0.01)		
Negative affectivity x Age x Medical clown	−0.02***	(0.00)		
*R*^*2*^	0.10			0.16

*p < 0.05, **p < 0.01, ***p < 0.001

## General discussion

In the current studies, we examined a practice commonly used to enhance health consumer satisfaction – the use of medical clowns in hospital wards. The objective of this research was to identify the optimal health care audience (adults or children) that will benefit most from medical clowns in elevating their satisfaction and thus reducing their aggressive tendencies. This is the first study to compare the two populations and to present a full scope perspective on the optimal placement fit of medical clowns from the point of view of the medical staff, the clowns themselves, and the health consumers. Studies 1 and 2 demonstrated that medical staff and clowns evaluated correctly that children health consumers profit from medical clowns more than do adults. They did not, however, recognize the possibility that clowns might have a negative effect on older adults. Accordingly, medical staff who recommended changes thought that more medical clowns would be generally useful in all wards. Clowns reported they are mostly placed in pediatric wards, adapting their performance to the health consumer type, mostly according to health consumer age and medical condition (which are often related). Even though they identify that their performance suits children better than adults, they would like to be placed in more hospital wards.

After collecting the perception of the medical staff and clowns perceptions about optimal placement, Study 3 continued to explore the actual optimal placement of medical clown, testing satisfaction antecedent and consequences following a visit by a medical clown.

Study 3 results indicate that medical clowns buffered the harmful influence of negative affectivity on satisfaction and aggression only among children and had a harmful opposite effect on older adults, suggesting that clowns are more useful when placed in children’s wards.

With respect to the first goal of Study 3, which was to determine the underlying mechanism that influences health consumers’ aggression towards medical staff, we found that negative affectivity has both a direct relationship with aggression as well as an indirect relationship, which is explained by health consumer satisfaction. Our findings are consistent with previous studies that found (a) a negative relationship between negative affectivity and satisfaction [43, 44, 61, 84], and (b) a positive relationship between negative affectivity and aggressive tendencies [58]. We extend these findings in our context and show that the mechanism by which negative affectivity influences aggression is by influencing satisfaction.

Concerning the second goal of Study 3, which was to examine the usefulness of medical clowns as an intervention aimed at reducing aggression, our study suggests that medical clowns moderate the relationship between negative affectivity and satisfaction, such that among health consumers who encounter a medical clown, the relationship between negative affectivity and satisfaction is weaker, thus indicating that medical clowns can buffer the harmful influence of negative affectivity on satisfaction. This relationship, however, depends on the age of the health consumers, and as our findings indicate, exists only for children and is reversed for older adults.

With respect to Study 3’s third goal, which was to determine the target health consumer audience that can most benefit from this intervention, our findings suggest that medical clowns are a useful method for increasing satisfaction and buffering aggression among children, but not among adults. Surprisingly, medical clowns had a negative effect on adults. Specifically, while in the absence of medical clowns, negative affectivity was not related to satisfaction or aggression, after adults encountered a medical clown, negative affectivity decreased satisfaction and increased aggression.

We propose that these results might stem from the general negative attitudes that adults might hold towards clowns. This can be illustrated using Lexical metaphors research (e.g., [85, 86]), which suggests that the metaphors we use in everyday life convey general perceptions and beliefs, which in turn influence social judgments and behaviors. The usage of common clown metaphors, such as “behaves like a clown” and “looks like a clown”, suggests that clowns are associated with behaviors that are considered childish, imprudent, and inappropriate for adults. As hospitalization is considered one of life’s most stressful events [87], it is reasonable to assume that adult health consumers are nervous and anxious, and expect to be treated in a manner they perceive as professional and serious. Therefore, if hospital health consumers associate medical clowns with behaviors they perceive as childish and irresponsible, the presence of a medical clown might elicit negative attitudes toward them and increase tension instead of easing it. Additionally, health consumers may feel that the use of clowns signifies un-adult-like treatment (i.e., not taking them seriously), which may also enhance negative feelings and decrease willingness to cooperate with medical clowns. These findings highlight the dangers of ignoring the possibility of age as a moderator.

To the best of our knowledge, no studies have yet tested the full model predicting hospital aggression, beginning with the main cause of dissatisfaction- negative affectivity, the mechanism of satisfaction, and the resulting health consumers’ aggressive tendencies toward hospital staff. Past research that examined health consumer satisfaction and aggression, is mostly descriptive and measured the phenomenon indirectly through staff self-report, rather than measuring aggression directly among the health consumers themselves (indirectly as reflecting their aggressive tendencies), as done in the current study [88, 89]. Moreover, no studies have examined the usefulness of specific interventions aimed at elevating health care satisfaction and reducing health consumers’ aggressive tendencies. In this research stream, we examined the usefulness of medical clowns in elevating satisfaction and buffering aggressive tendencies among health consumers of different ages, in a multiple views research stream.

This research contributes to practice by identifying the optimal placement of medical clowns - children’s wards. It contributes to theory by revealing the mechanism of (dis) satisfaction, by which medical clowns can buffer aggression stemming from negative affectivity.

### Limitations and future directions

This study has several limitations. First, the reliability of our satisfaction measure was low (0.67), although it was higher than the reliability of other satisfaction measures [90]. Additionally, we used self-report measures of aggression; future studies should seek to use behavioral measures. Third, the sample is not entirely random, but rather a convenience sample, even we tried to simulate randomization as much as possible by sampling the health consumers at random times of the day and week, thus partially circumventing the limitation of randomness.

Future research should continue to examine the usefulness of medical clowns among adults and examine whether medical clowns do have a positive effect on adults in other aspects that are beyond the scope of the current work. One possible explanation for our finding that medical clowns benefit children but may detrimentally affect adults, is that the current services provided by medical clowns are not adapted uniquely to adults, and the clowns may simply lack correct guidance in order to provide a suitable approach for amusing adults. Future research should examine the practices used by medical clowns on the different audiences, and determine whether using a tailored approach may elevate their satisfaction. Other moderating variables can be explored that might add more complexity to the boundary condition of the effect beyond the age of the health consumer, this might include clown’s characteristics, such as individual differences in the need to possess high emotional intelligence [91] and the ability to be relationally present with the elderly [9].

In addition, future research can examine additional methods, other than medical clowns, for mitigating the harmful influence of negative affectivity on aggression via satisfaction, by finding other ways to enhance satisfaction among adult health consumers.

## Conclusion

In this paper, we identify the optimal audience that will most benefit from medical clowns, namely children, as well as the audience that will least benefit from medical clowns, namely older adults. These findings are especially important since medical staff did not fully recognize the different effects medical clowns have on health consumers of different ages. Our findings can guide policymakers by indicating the optimal placement of clowns, i.e. in pediatric wards, thereby benefitting most from the clowns’ efforts, elevating health consumer satisfaction, and reducing aggressive tendencies.

## Supplementary Information

**Additional file 1.**

**Additional file 2.**

**Additional file 3.**

## Data Availability

Data can be found at: https://osf.io/8j7ar/?view_only=c0cd6308be634b948235132df01612bd
