# Supplementary material for: Exploring the usefulness of medical clowns in elevating satisfaction and reducing aggressive tendencies in pediatric and adult hospital wards
Source: BMC Health Serv Res. 2021 Jan 6;21:15. doi: 10.1186/s12913-020-05987-9 (PMC7789247; doi:10.1186/s12913-020-05987-9)
Supplement: Supplementary file 1 — Additional file 1. [file 12913_2020_5987_MOESM1_ESM.docx]

**Additional File 1**

**Study 1a Survey**

**Participants: Medical Staff**

***Current placement policy***

Is there a medical clown in your hospital? _____

If yes, in which ward(s) is the medical clown placed? ________________________________

***Perceived optimal placement policy***

What is your opinion about the current ward where the medical clowns are placed in your hospital?

1 - no change to placement policy needed;

2 - change the placement policy so that they are placed more frequently in adult wards;

3 - change the placement policy so that they are placed more frequently in pediatric wards;

4 - change the placement policy so that clowns are generally placed more frequently, in all wards;

5 - change the placement policy so that clowns are placed less frequently in general.

***Perceived health consumer satisfaction with the clowns*** was measured using 7-point Likert-type scale. Survey is based on Wong (66)‏:

In your opinion, to what extent are most adults: (1- not at all; to 7- very much)

1 - Satisfied with the clown’s performance

2 - Happy with the clown’s performance

In your opinion, to what extent are most children: (1- not at all; to 7- very much)

1 - Satisfied with the clown’s performance

2 - Happy with the clown’s performance

***Demographics***

Age: ____

Gender: _____

Job title: _____

Tenure in current job: ______
