# Supplementary material for: Exploring the usefulness of medical clowns in elevating satisfaction and reducing aggressive tendencies in pediatric and adult hospital wards
Source: BMC Health Serv Res. 2021 Jan 6;21:15. doi: 10.1186/s12913-020-05987-9 (PMC7789247; doi:10.1186/s12913-020-05987-9)
Supplement: Supplementary file 3 — Additional file 3. [file 12913_2020_5987_MOESM3_ESM.docx]

**Survey for patients and escorts in Haemek Hospital**

Hello,

We invite to you to participate in a study that examines the feelings, experiences and the quality of service you receive at the hospital. The survey is anonymous.

Do you agree to participate in the study and answer the questionnaire (circle) yes / no

We thank you in advance for your participation in the study. In the following questions we would like to learn about your own opinion. There is no ' correct ' or ' incorrect ' answer to any of the questions. All your answers are anonymous and will be kept confidential.

1. In which ward are being treated? _________________
2. I am in (circle) daily hospitalization/full hospitalization

|  | Very unsatisfied | unsatisfied | neutral | satisfied | Very satisfied |
| --- | --- | --- | --- | --- | --- |
| 1. How satisfied are you with the medical treatment? | 1 | 2 | 3 | 4 | 5 |
| 1. How satisfied are you with the physical conditions in the hospital? | 1 | 2 | 3 | 4 | 5 |
| 1. 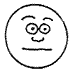 Circle the face that best represents how you feel now | 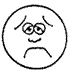 | 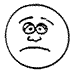 |  |  | 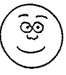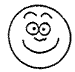 |

**What is the likelihood that in the near future someone in this ward will….**

|  | Very low | Pretty low | low | medium | high | Pretty high | Very high |
| --- | --- | --- | --- | --- | --- | --- | --- |
| 1. curse a staff member | 1 | 2 | 3 | 4 | 5 | 6 | 7 |
| 1. behave nicely towards a staff member | 1 | 2 | 3 | 4 | 5 | 6 | 7 |
| 1. hit a staff member | 1 | 2 | 3 | 4 | 5 | 6 | 7 |
| 1. push a staff member | 1 | 2 | 3 | 4 | 5 | 6 | 7 |
| 1. speak politely toward a staff member | 1 | 2 | 3 | 4 | 5 | 6 | 7 |
| 1. damaging hospital property | 1 | 2 | 3 | 4 | 5 | 6 | 7 |
| 1. pound on the table | 1 | 2 | 3 | 4 | 5 | 6 | 7 |
| 1. insult a staff member | 1 | 2 | 3 | 4 | 5 | 6 | 7 |
| 1. behave honorably toward a staff member | 1 | 2 | 3 | 4 | 5 | 6 | 7 |
| 1. yell at a staff member | 1 | 2 | 3 | 4 | 5 | 6 | 7 |
| 1. complain about the hospital | 1 | 2 | 3 | 4 | 5 | 6 | 7 |
| 1. report the staff member to the hospital management | 1 | 2 | 3 | 4 | 5 | 6 | 7 |
| 1. complain about a staff member to the hospital management | 1 | 2 | 3 | 4 | 5 | 6 | 7 |
| 1. offend the staff member’s family publicly | 1 | 2 | 3 | 4 | 5 | 6 | 7 |

1. **During your visit to the hospital did you meet a medical clown? If so, when? Please circle the correct answer**

| 1 | 2 | 3 | 4 | 5 | 6 | 7 |
| --- | --- | --- | --- | --- | --- | --- |
| In the past hour | A few hours ago | yesterday | Two days ago | This week | More than a week ago | Never |

**To what extent do you agree with the following sentences?**

|  | Not at all | hardly | Very little | Moderately | slightly | much | Very much |
| --- | --- | --- | --- | --- | --- | --- | --- |
| 1. Before criticizing somebody, I try to imagine how I would feel if I were in their place. | 1 | 2 | 3 | 4 | 5 | 6 | 7 |
| 1. If I'm sure I'm right about something, I don't waste much time listening to other people's arguments | 1 | 2 | 3 | 4 | 5 | 6 | 7 |
| 1. I sometimes try to understand my friends better by imagining how things look from their perspective. | 1 | 2 | 3 | 4 | 5 | 6 | 7 |
| 1. I believe that there are two sides to every question and try to look at them both. | 1 | 2 | 3 | 4 | 5 | 6 | 7 |
| 1. I sometimes find it difficult to see things from the "other guy's" point of view | 1 | 2 | 3 | 4 | 5 | 6 | 7 |
| 1. I try to look at everybody's side of a disagreement before I make a decision. | 1 | 2 | 3 | 4 | 5 | 6 | 7 |
| 1. When I'm upset at someone, I usually try to "put myself in his shoes" for a while. | 1 | 2 | 3 | 4 | 5 | 6 | 7 |
| 1. I've been concentrating my efforts on doing something about the situation I'm in. | 1 | 2 | 3 | 4 | 5 | 6 | 7 |
| 1. I've been taking action to try to make the situation better. | 1 | 2 | 3 | 4 | 5 | 6 | 7 |
| 1. I've been trying to come up with a strategy about what to do | 1 | 2 | 3 | 4 | 5 | 6 | 7 |
| 1. I've been thinking hard about what steps to take | 1 | 2 | 3 | 4 | 5 | 6 | 7 |
| 1. I've been making jokes about the situation. | 1 | 2 | 3 | 4 | 5 | 6 | 7 |
| 1. I've been making fun of the situation | 1 | 2 | 3 | 4 | 5 | 6 | 7 |
| 1. I've been trying to find comfort in my religion or spiritual beliefs | 1 | 2 | 3 | 4 | 5 | 6 | 7 |
| 1. I've been praying or meditating. | 1 | 2 | 3 | 4 | 5 | 6 | 7 |
| 1. I've been getting emotional support from others | 1 | 2 | 3 | 4 | 5 | 6 | 7 |
| 1. I've been getting comfort and understanding from someone | 1 | 2 | 3 | 4 | 5 | 6 | 7 |
| 1. I've been turning to work or other activities to take my mind off things. | 1 | 2 | 3 | 4 | 5 | 6 | 7 |
| 1. I've been doing something to think about it less, such as going to movies, watching TV, reading, daydreaming, sleeping, or shopping | 1 | 2 | 3 | 4 | 5 | 6 | 7 |

**It is important for us to know how people feel about our study, in order to evaluate the quality of our work. Please answer the following questions by choosing the most appropriate response in your opinion:**

|  | Not at all | hardly | Very little | Moderately | slightly | much | Very much |
| --- | --- | --- | --- | --- | --- | --- | --- |
| 1. How professional are the questions you have been asked until now? | 1 | 2 | 3 | 4 | 5 | 6 | 7 |
| 1. How clear are the questions you have been asked until now? | 1 | 2 | 3 | 4 | 5 | 6 | 7 |
| 1. How vague are the questions you have been asked until now? | 1 | 2 | 3 | 4 | 5 | 6 | 7 |
| 1. To what extent are the questions written in a high level? | 1 | 2 | 3 | 4 | 5 | 6 | 7 |
| 1. How professional are the researchers who built the survey, in your opinion? | 1 | 2 | 3 | 4 | 5 | 6 | 7 |
| 1. How amateur are the researchers who built the survey, in your opinion? | 1 | 2 | 3 | 4 | 5 | 6 | 7 |

**We would like to know a little about the people who participated in the study. All information you provide will be kept confidential and will not be transferred to anyone.**

1. Age ____
2. Gender (circle) male/female
3. Education level (grade you have graduated) ____
4. Number of rooms in your home ____
5. Number of people who live in your home _____
6. Religion (circle) Jewish/Muslim/Christian/Druze/other, please specify _____
7. I consider myself (circle ) secular / traditional / religious / very religious / other, please specify__
8. My mother tongue is ( circle ) Hebrew / Arabic / Russian / English / European language / Ethiopian / other, please specify _____

**Thank you for your participation!**
